# The Algoid Origin of Miasmatic Fevers

**Published:** 1868-03

**Authors:** 


*The Algoid Origin of Miasmatic Fevers.*—We have already alluded to Prof. Salisbury's experiments and observations upon this subject. We notice that they are attracting attention on the other side of the Atlantic. The Lancet gives us an outline of the researches of Dr. Heinrich Schmidt, who studied the subject during the recent frightful epidemic in the Mauritius. In persons dying with fever, this gentleman found microscopic plants like the *Cryptococcos cerevisiae* covering the lining membrane of the stomach. He was also able to detect them in the secretions of the corners of the mouth, of the tongue, and even upon the surface of the skin. He found allied forms in the water taken from the pools near the Grand River, and also from the sea at the mouth of that stream.

Another writer states that when examining the fructification of algœ, he was warned by his preceptor to be careful at the time of their fructification, as they would certainly give him intermittent fever. He thought however, that, as he was growing his plants in pure water remote from their marshy home, he might venture to disregard the warning. At the time they were throwing off the spores, he was nevertheless prostrated by a severe attack of intermittent fever.

